# Roadmap for rebuilding the health system and scenarios of crisis path in Gaza

**DOI:** 10.1002/hpm.3861

**Published:** 2024-10-16

**Authors:** Mohammed Alkhaldi, Malak Alrubaie

**Affiliations:** ^1^ Faculty of Communication, Arts and Sciences Department of Public Health Canadian University Dubai Dubai UAE; ^2^ Faculty of Medicine School of Physical and Occupational Therapy McGill University Montreal Quebec Canada; ^3^ Nuffield Department of Medicine Centre for Tropical Medicine and Global Health University of Oxford Oxford UK; ^4^ Faculty of Health University of Waterloo Waterloo Ontario Canada

**Keywords:** crisis scenarios, Gaza crisis, health system recovery and rebuilding, Palestine

## Abstract

The horrific attacks on Gaza have had a profound impact on Gaza's health system, culminating in a multidimensional crisis. The deliberate destruction of vital infrastructure, such as hospitals, schools, housing, and public facilities, coupled with the deaths and injuries of medical personnel and support workers has only exacerbated the situation and further highlighted the existing gaps. This unprecedented catastrophe proves the criticality of adopting a new national inclusive integrated approach to meeting the immediate and long‐term needs of the population. In this perspective, we explore the recovery roadmap features for rebuilding the health system in Gaza, specifically focusing on determining the primary challenges that might emerge, the trajectory of recovery, and the expected crisis scenarios. The existing evidence and perspectives of key stakeholders, including state and non‐state health authorities in Palestine were synthesised. Despite some local and international initiatives undertaken to generate a concrete road to recovery, there remains a need for realistic, innovative, and comprehensive Marshall plans to rebuild Gaza's health system. The article draws on insights and gaps in current efforts and underscores the urgent need to address the challenges of rebuilding the health system. The authors strive to offer an inclusive and realistic path with the potential scenarios toward recovery and resilience considering the mass levels of loss and damage, and ways to move forward for building back a resilient health system in Gaza.

## Highlights

- During the 12 months of the ongoing crisis in Gaza, the severely attacked and damaged health system leaves the majority of the population deprived of essential healthcare services and marginalised to huge health threats.
- Rethinking the health system and adopting a new and strategic national inclusive integrated approach with a clear national rebuilding health system to meet the immediate and long‐term needs of the population is imperative.
- Tackling the profound humanitarian challenges and essential life‐saving needs taking into account the scenarios of the crisis path is highly needed to effectively rebuild the health system.
- As the way forward, steadfast political commitment from all stakeholders backed by different and more effective approaches and mechanisms of collaboration, transparency, and accountability are central in the face of the 21st century's deadliest crisis with a focus on the restoration of population lasting health and dignity and realisation of the rights‐based and health‐peace approaches and ending the lengthy injustice.

## 1 INTRODUCTION

The crisis in Gaza demonstrates the vast magnitude and immense lasting adverse effects on people's lives and all social systems. To present, 1.7 million people are displaced several times and are on the move again due to military operations and Israeli evacuation orders, with at least 36,479 Palestinians have been reportedly killed since 7 October, another 82,777 injured, and hundreds were detained. This scene is exacerbated by the spread of diseases, starvation, and the vast level of destruction of all public facilities and residential buildings, which constituted more than 70%, in addition to the huge damage to vital infrastructure and systems, including the health system.^1^ Following the imposition of a tight siege on Gaza in 2007, Israel's systematic actions and policies have consistently damaged and overwhelmed the health system in Gaza as well as throughout the Palestinian territory.^2^ Currently, the current Israeli aggressive and catastrophic military measures on Gaza have rendered the fragile health system inoperable; only 16 of Gaza's 36 hospitals remain partially functional.^1^ It is confusing where and how to begin examining and analysing this unprecedented and compound disaster which may amount to a genocidal act. It is also highly uncertain to envision approaches and ways for recovery and rebuilding the health system in Gaza while its pillars are largely broken amidst the continuity of large‐scale killing, damage, and scarcity.

This perspective explores innovative recovery approaches for rebuilding Gaza's resilient and responsive health system. The viewpoint employs a grounded approach, integrating a thorough review of evidence and synthesising perspectives from diverse local and international stakeholders involved in the Palestinian health system, including the Ministry of Health, local and international non‐governmental organisations, and the academic sector. The insights presented in this viewpoint synthesised from the existing evidence and stakeholders' perspectives were also enriched by the authors' insights. This would aid local and international health and humanitarian actors and policymakers during and after the crisis. We focused on three critical questions to help envision the recovery and rebuilding roadmap and process. The first question is what the primary public health problems and challenges that health system rebuilding may face. The second question is what elements and trajectories of recovery need to be considered in Gaza's post‐crisis rebuilding process. The third and final question is what a self‐determined recovery process and scenarios might be anticipated for the health system in Gaza. These scenarios of the crisis path inform all local and international health policymakers and professionals about the recovery challenges and insights, allowing for better thinking, planning, and intervention.

### 1.1 Challenges of recovery

The first step that must be a priority of all local and international state and non‐state actors is to address the most pressing life‐saving issues/needs that all Palestinians in Gaza profoundly lack. The immense concern is livability and survival, which may appear challenging in this little enclave. After the 2007 siege was imposed,^2^ Gaza was called a ‘ghetto’ and ‘the world's biggest open‐air prison’ surrounded by multilayered barriers.^3^ Unfortunately, with the emergence of the devastating crisis, it is now being referred to as ‘the world's biggest graveyard’.^4^ It is not only a graveyard for people, but also for humanitarian laws and principles. Despite all the heartbreaking scenes and stories, history taught us that rights holders and freedom seekers can recover, rise, and stand up after extreme adversities. Indeed, the Palestinians and Gazans, in particular, are able to recover and stand up again amidst annihilation, as they have always done for the past 75 years. All actors must direct their primary efforts on addressing the challenges of building shelters in safe areas for people, rebuilding their homes and infrastructure, and providing food, electricity, safe water, sanitation and roads. These challenges are deemed by the UN the most essential for survival and public health priorities.^5^ However, there is a limitation in precisely recognising the public health problems and challenges in this unique humanitarian setting as no systems or infrastructure are left in place. The mass destruction of the health system is the biggest problem in itself. Addressing this limitation through a thematic and exploratory analysis based on existing literature, experts' perspectives, and authors' insights is the most suitable approach.

Additionally, application of similar conventional recovery models and approaches in the aftermath of Gaza's crisis should be considered. There is now a golden opportunity to utilise new models and approaches to rebuild and re‐design the entire health system from scratch, as the radical destruction caused by the crisis has created the need for a fundamental transformation that goes beyond simply restoring the previous model. Rebuilding and recovering Gaza's health system could be a key step for a future comprehensive health system in Palestine. Enhancing the health system recovery and other social systems demand a multi‐stage strategy, necessitating proper knowledge, expertise, planning, and funding and alignment with socio‐political interests. The absence of any of these critical components poses formidable challenges to rebuilding and recovery. Addressing the immense impact of the disaster is a priority before any reconstruction. This process will be lengthy considering the extensive loss and damage throughout the restoration of healthcare operations, including service delivery.

Moreover, the coordination body and mechanism that were not been sufficiently effective before the crisis is problematic. Such body and mechanism should be inclusive to representing all stakeholders and disciplines relevant to recovery, primarily civil society. An additional consideration on the traumatised skilled human resources, workers, and leaders in healthcare is required. Therefore, the recovery process must include support for these health workforces and recruiting new staff to initiate this process. In other words, the process that does not consider the severity of human losses and infrastructure damage of Gaza's health system will undoubtedly be ineffective. Rebuilding all of Gaza's systems, sectors, and facilities would require exuberant amounts of human, financial, and material resources, which have been destroyed. The health system, for instance, has lost at least 484 healthcare workers and around 435 healthcare facilities attacked.^1^

As outlined earlier, the severe shortage of the health and humanitarian workers due to murder, detention, or injury, damage of basic infrastructure, and scarcity of financial resources make it difficult for the Palestinians to restore the health system better than before. Accordingly, a comprehensive Marshall recovery and feasible rebuilding plan that deals with this draconian situation essentially requires a knowledge‐based comprehensive assessment that guides all stakeholders' plans. Such robust and comprehensive assessments and their results were not effectively carried out and translated in previous crises. The current crisis is more than just health‐related; it affects everything and it has both direct disastrous impacts on health as well as on public health, resulting in various demographics, including men, women, and individuals of different age groups.^6^ The numerous casualties have resulted in a significant increase in traumatic injuries, such as amputations, further straining an already overwhelmed health system.^7^ As a result of performing this assessment and prioritisation assignment, we will clearly understand the extent of difficulties being faced. This assignment should take into account public health issues (such as lack of healthcare infrastructure and resources, access to essential services, and resources for preventive care and mental health needs and trauma), environmental disruption, and long‐term impact. The basic needs, such as housing, food, and clean water should be also included. The absence of these comprehensive assessments hinders all prioritisation, planning, and rebuilding actions, and all Palestinian health, education, environment, and development sectors should be involved to supporting the health system. Generally, all these health, public health, and health system‐related problems/challenges need to be recognised by all stakeholders in the discourse and plans for health system recovery and rebuilding.

This perspective identifies eight challenges and it informs the Palestinian and international actors and decision‐makers on how they can address these challenges now and during the planning and implementation of the recovery plan. These challenges are:

1. The Magnitude of human losses and damage to infrastructure.
2. The mental health crisis.
3. Outbreaks and mortality rates of communicable and non‐communicable diseases.
4. Shortage in the health workforce.
5. Collapse of the public health system and inaccessibility to sanitation and clean water, unhealthy living conditions, and preventive, promotive, and protective services.
6. Food insecurity, starvation, and malnutrition.
7. Shift from emergency to recovery with the politicisation and securitisation of humanitarian aid and recovery plans.
8. Disrupted governance structure and health system functions.

First, the mass magnitude of human losses and damage that resulted interruptions of all public services, including healthcare, at all sectors and all Gaza areas is a key challenge.^8^ The health system in Gaza has significant damage in medical equipment, assets, supplies, and facilities at all MOH and non‐state health settings.^8^, ^9^ Furthermore, essential public infrastructure such as roads, cities, villages, and community facilities supporting public health services, including water supply, wastewater management, sewage systems, solid waste management, and roads, have been also damaged. Second, the mental health crisis leaves a significant number of the population suffering from constant trauma and psychological disorders (depression, anxiety, and post‐traumatic stress disorder) as a result of intensive and aggressive violence.^6^ Third, the noticeable rise in the morbidity and mortality rates of communicable and non‐communicable diseases emerged due to the destruction of systems, lack of good healthcare, food, and water, and overcrowded and unhygienic shelters.^7^ Fourth, the health workforce shortage, which has deepened as a result of death, emigration, injury, displacement, evacuation, detention, and interrupted medical education complicates the recovery process. Restoring the health system services requires recruiting and training new workforce and supporting services to address the physical and emotional needs of existing staff. Fifth, poor sanitation, large amounts of solid waste, limited access to clean water, poor living conditions, and collapsed public health preventive, promotive, and protective services pose a major concern. Sixth, large‐scale food insecurity, starvation, malnutrition, and the effects have become prevalent among children, women, and the elderly due to the tied restrictions of aid flow is also an expected intricating issue.^6^, ^7^, ^10^ The shift from emergency to recovery including the politicisation and securitisation of humanitarian aid and support and the multiplicity of actors is another challenge.^11^ The political and security pressures underestimate Palestinian autonomy in light of the huge population's demands. This is expected to influence the proper implementation of recovery and rebuilding plans. The eighth and final expected challenge is the disrupted governance system and its main functions. This disruption exacerbates issues of control, leadership, communication, financing, and planning of all health system facilities, teams, and networks.

Before this crisis, Gaza's healthcare system struggled to function properly due to long‐standing blockades and restrictions.^11^ However, demolition of road networks and residential areas throughout Gaza has further limited access to health facilities. Health authorities' capacity in resilience, recovery, rescue, and preparedness has been severely weakened due to the constant target of health system infrastructure. This situation has led to limiting the system's ability and essential services such as disease prevention and surveillance and disaster management and response due to the disrupted communication and transportation. These governance‐related challenges hindered the effective system responsiveness to the crisis and the population's health needs. As a result, Gaza's health system remains ill‐equipped to cope with this unprecedented scale of the current crisis. There is an urgent need investment in rebuilding resilient health system infrastructure and preparedness mechanisms.

### 1.2 Trajectories of recovery

Building on the above‐identified problems and referring to a plan for Europe's economic recovery after World War II, a new national Marshall recovery plan that includes a health system recovery in Gaza with strategic and game‐changing trajectories. The UNDP's initial estimates for the reconstruction of Gaza surpass $30 billion and could reach up to $40 billion and it would take decades.^12^ However, experts estimated that rebuilding the damaged infrastructure of the health system will take at least 10 years and $15 billion during the current war.^13^ The significance of this analysis stems from the urgent necessity of thinking of and framing this plan which can already begin, even amid ongoing war conflict to identify the pressing needs and priorities. The proposed financing facility for the Marshall Plan for Gaza is expected to establish a mixture but unified and integrated local and international financing mechanisms managed by an executive and advisory well‐representative board of this facility. Overall, this plan that includes a health system rebuilding plan should compass three stages: (1) immediate comprehensive needs assessment and humanitarian aid and medical supplies and support, (2) rebuilding the livability and survival of people, and (3) rebuilding all public social systems, including rebuilding a comprehensive health system and services.

This plan might be sustained for at least 10 years and reconstructing the healthcare system in Gaza should be aligned with WHO standards and integrated into other sectors' national recovery efforts.

In the first stage of the plan, which might last one to 2 years, must begin by conducting a national comprehensive needs assessment and prioritisation. Health system actors must execute this exercise immediately to identify the actual health system and population's public health needs, which include capacities and resources for the health system and injuries, malnutrition, infectious disease, and mental health issues for the population. WHO along with international bodies including the World Bank, UN agencies, Oxfam, Save the Children, MSF, and MAP‐UK can provide support to Palestinians in carrying out this assessment through international and local Joint External Evaluation (JEE) and apply various useful tools such as the WHO's Strategic Tool for Assessing Risks (STAR) and the World Bank's Crisis Preparedness and Response Toolkit.^14^, ^15^

This is supposed to be followed by carrying out a technical health system analysis that covers the following elements: health policy and planning, governance, workforce, financing, infrastructure and logistics (buildings, supplies), and healthcare and public health services (primary, secondary, tertiary, disease control, sanitation). Moreover, mobilising both international humanitarian and medical support should be urgently supplied in Gaza. The immediate humanitarian and relief needs such as food, water, shelter, fuel, and medical and hygienic supplies are enormous and require secure corridors and coordinated actions that go well beyond the health sector. Importantly, specialised medical teams and medical supplies are necessary, especially now and in the early phases of the recovery plan. This support would guarantee the availability of medicine and medical supplies and the sufficiency of the healthcare workforce that covers all medical needs. Addressing the depletion of the healthcare workforce by providing training and support for existing staff, attracting healthcare workers back to the area, and prioritising the psychosocial well‐being of healthcare professionals who may have been traumatised is a priority.^16^ An international and regional network of hospitals can be established to evacuate the huge number of wounded people to neighbouring countries. This action has to be part of the recovery plan during the current crisis and post‐crisis with full consideration of main medical and surgical subspecialities.

The subsequent immediate and mid‐term actions that the recovery plan must also receive greater attention is providing primary health care through mobile clinics and outreach health teams in certain areas. In the secondary healthcare provision, field hospitals with large bed capacity should be deployed in all of Gaza's Governorates with the importance of effective and unified referral mechanisms for patients outside or inside Gaza. Re‐engineering, re‐designing, and re‐allocating the primary, secondary, and tertiary healthcare services may provide an opportunity to rebuild stronger healthcare delivery models. However, the long‐term reconstruction phase will require rebuilding and rehabilitation of entirely and partially the damaged health system and its infrastructure and facilities such as the main hospitals, primary health centres, laboratories, and other public health facilities, and equipping them with the necessary equipment and capacity. Immediate actions will also focus on public health through vaccinations, communicable and non‐communicable diseases, maternal and child health, nutrition, mental health, supply of water, and environmental health services. The recovery plan would ensure access of all populations, especially patients and groups at risk to essential medical and public health services to stop and control health complications, high morbidity and death rates, and environmental threats.

The second immediate and short‐term action is the provision of the basics for the livability and survival of people. This action is an essential action for the sake of guaranteeing people's survival, health security and protection and then rebuilding the health system. Rebuilding or restoration of people's resilience and normality through immediate first aid and support are key and should proceed with rebuilding the health system and its facilities and services which are gradually built based on the highest priorities and in a comprehensive and integrated recovery plan. Rebuilding the public health system and healthcare services entails more than just reconstructing hospitals, health centres, and services, it goes beyond that. This approach is helpful, and it must first begin with the modest restoration of people's daily living conditions and going back to normal lives such as going back to work, school, and so on. Utilising and combining both approaches, with a focus on building back normal life and vital social systems and the health system simultaneously is essential. This integration of recovery and rebuilding approaches could make impacts in the short and long‐term. However, the integrative plan would take at least 10 years because it is a matter of rebuilding other vital systems and sectors such as the energy, communication, economy, water and sanitation, housing, agriculture, education and academic systems including health. For instance, the education system can support bridging the workforce shortage gap by resourcing the health system with trained physicians, nurses, and allied health professionals. The recovery and rebuilding plan of the health system requires a consolidated strategy and intersectoral implementation.

Furthermore, in this second stage, psychological and social support is central and demanded. Rebuilding the liveability and survivability of the population and the society restores the psychosocial patterns and social lives, assisting those affected, particularly children, the elderly, and women, in resuming their everyday lives as soon as possible. Since health is an integral part of the social paradigm, a recovery plan should also pay more attention to particular populations such as children who have lost parents, grandparents, or all family members, and people with disabilities. These issues are important and local and international actors need to design long‐term, inclusive, and integrated social and rehabilitation programs that target those populations and wounded Palestinians transferred abroad for special treatment. In general, there is a need for a comprehensive approach toward addressing the root causes of health issues such as poverty, unemployment, and food insecurity through evidence‐based interventions.

The third stage of the recovery plan which is rebuilding the central systems including the health system could be the toughest task. The reason behind this difficult task is that rebuilding all public social systems, including rebuilding a comprehensive health system and services is a strategic and collective exercise and it entails long‐term actions and immense resources and capacities. In this stage, there will also be chances to add components that were not available before. Setting priorities and following a certain order are therefore essential components in rebuilding the health system in Gaza, especially as choices made during the transition phase will frequently dictate the system's long‐term course of development. Among these rebuilding priorities are limited financing, a shortage of main subspecialities and human resources, and a broken health information system to enhance the monitoring of performance and outcome indicators. However, the recovery of the health system after the crisis needs to be defined and contextualised to match the local context. The WHO building blocks model, for example, is broad and places more emphasis on the supply than the demand side of the health system.^12^ Prioritising patient‐centred measures in health system reintegration should involve considering factors including acceptability, security, accessibility, and geography—particularly in situations where there has been widespread relocation. However, since non‐state organisations and community‐based organisations have a lot of power, it is equally important to guarantee community engagement.^17^, ^18^

The resiliency of the health system should be considered in this recovery stage of rebuilding the health system. Recovery initiatives, which are associated with the resilience of the health system, usually start during the emergency period and build upon humanitarian successes to launch sustainable development processes. Furthermore, the plan requires embracing the ‘build back better’ concept, which calls for reconstructing the system in a way that leads to it being more sophisticated than it was before the catastrophe.^18^ Most importantly, the sustainability factor is imperative in the recovery plan to rebuild a sustainable health system that can function in the long term. This can be achieved in this plan by building local capacity, developing a funding mechanism supported by the local economy, and addressing pre‐existing weaknesses in the healthcare system that the disaster may have exacerbated.

The third stage of the recovery and rebuilding plan of the health system should also consider strengthening emergency preparedness and institutionalising intersectoral partnerships and integration with governmental, NGOs, and other sectors during and post‐crisis. Better preparedness and integration across sectors support rebuilding and implementing effective and consolidated health strategies that secure, for instance, essential drugs and medical supplies, prevention and control of emerging diseases, and WASH programs.

All stakeholders involved in this stage such as Palestinian Ministries, civil society, private sectors, and international actors should take into account another critical trajectory there is related to the management of the rubble and debris from the destruction in Gaza and its long‐term dangerous effects on the environment, animal, and human. It is also important that the stakeholders consider handling unburied bodies and collective graves. Post‐conflict demolition waste management processes must include access to debris for collection and transportation to remove and recycle the materials that comprise the majority of rubble concrete. It is critical to thoroughly separate the various waste items, such as hazardous elements, to efficiently and effectively be able to recycle and reuse those materials that could be reused in the rebuilding process. Concerning the sensitive issue of handling the unburied bodies and mass graves stuck in the destruction, careful documentation, identification, and respectful treatment would be imperative. As part of the plan, it is crucial to stress that multidisciplinary committees and teams including public health, legal, and religious, are required to manage both the debris and the unburied bodies from the disaster.

On top of all the trajectories of recovery outlined, community engagement is the final but the most important driving force for the successful implementation of the recovery and rebuilding plan for the health system in Gaza. Engaging community leaders and representatives, initiating constant dialogues with residents to gather feedback and active involvement of community‐based organisations in decision‐making processes make this plan feasible and real.^18^, ^19^ More importantly, emphasising the importance of decolonising the Gaza context and Palestinians' self‐determination and autonomy, the processes of this recovery and rebuilding plan should be managed and controlled by both Palestinian institutions and community and civil society with the support of international actors and without external influence. It is crucial to stress that a robust, representative, and inclusive coordinating body or mechanism is required. Ensuring ownership and stewardship of Palestinian institutions as a part of this plan would help to establish a culturally acceptable and respectful approach that facilitates seamless implementation of the plan.^15^ Generally, to ensure all these stages are properly done, pre‐requite political factors such as agenda, interest, and priorities alignment, an independent, transparent, and accountable governance body that involves all stakeholders, especially the civil society organisation, and a fair monitoring mechanism should be established. Technically, national coordinated, integrated, and collaborative implementation plans across Palestinian sectors, international organisations, NGOs and the community are crucial to execute the plan and address such complex interrelated issues effectively.

### 1.3 Possible scenarios

With Gaza in ruins, millions displaced, and an uncertain future looming, the pressing question of what lies ahead remains outstanding. The implementation of this recovery plan for the health system depends on three possible scenarios. These scenarios can be analysed to better comprehend and navigate the rebuilding of Gaza's health system. Firstly, the examination of the potential outcomes if the current situation persists. Secondly, the ramifications of the implementation of a temporary ceasefire. Thirdly, the most favourable scenario is the exploration of the potential impact of a permanent lasting ceasefire. In shaping these scenarios, it is imperative to acknowledge the increasing significance of governance and politics, as these variables will without a doubt influence the course of Gaza's health system recovery.

The proposed scenarios align closely with those outlined in the Scenario‐based Health Impact Projections Report, emphasising the urgent need for a comprehensive health response. To conclude, across all scenarios, there is a notable increase in mortality rates. Using the widely recognised crude death rate metric among humanitarian actors, mortality is projected to escalate to 0.34, 1.70, and 2.16 deaths per 10,000 person‐days under the ceasefire, status quo, and escalation scenarios, respectively.^7^

#### 1.3.1 Scenario 1: The current situation persists

In this scenario, the health system in Gaza will deteriorate further, exacerbating the humanitarian catastrophe and making it increasingly challenging to provide the necessary services and needs of the population. Should the conflict persist, whatever little existing infrastructure and capacity, almost 30%, that remains standing will undoubtedly be further implicated. This is especially true in the north governorates of Gaza. If this scenario happens, it will make it extremely difficult to rebuild the health system and adopt effective and sustainable restoration solutions due to the continued systematic and large‐scale destruction and vast casualties. People will continue to lack access to clean water, sanitation, and hygiene facilities due to Inadequate shelter and WASH facilities. Food security and nutritional status will also continue to be compromised, which will exacerbate malnutrition and related health issues. Furthermore, the healthcare system will be severely overburdened as a result of the rising rates of trauma, injuries, infectious disease, and casualties. The Palestinian population will continue to suffer substantially from physical and psychological trauma.

To address these challenges, immediate international support and advocacy efforts are essential to seek a permanent ceasefire and facilitate access to aid. Establishing a safe, sustainable and seamless supply mechanism of medical and humanitarian assistance is imperative to maintain the minimum functionality of the health system and to address immediate needs such as food, water, and shelter. This mechanism supposed to be managed by the UN agencies is essential and it is supported by other local and international efforts to protect the remaining healthcare facilities and ensure the provision of emergency medical services. This scenario is characterised by more uncertainty and higher levels of deterioration and escalations. It imposes the necessity of sustaining and mobilising all resources and coordinating interventions among stakeholders with a focus on the protection measures for healthcare workers. More importantly, strengthening public health surveillance must also be taken into consideration. This action aids health institutions in monitoring, responding, and detecting the expected waves of disease outbreaks. However, global health accountability is required and this global community, especially powerful countries and actors of the G7 and G20 along with the UN bodies, are required to use their power and diplomacy to pressure towards immediate sustainable solutions to end this local crisis that is becoming a worrying global health issue.^20^

#### 1.3.2 Scenario 2: The implementation of a temporary ceasefire

In this scenario, a temporary ceasefire would open up a brief window of opportunity for humanitarian relief and the delivery of critical supplies to Gaza's affected populations. However, this would only be contingent on the terms and conditions that relate to the ceasefire agreement. While this scenario would likely lead to a reduction in casualties and impairments, it would not address the underlying causes of the conflict, leaving Gaza's health system at risk of future disruptions. Displacement and overcrowding will somewhat decrease, but the underlying issues will not be resolved. People will still be unable to obtain clean water, sanitary conditions, and hygiene supplies due to Inadequate shelter and WASH facilities. Although there will be a minor improvement in food security and nutritional status, the blockade will nonetheless restrict access to essential goods. A ceasefire will reduce the number of casualties, however insufficient medical supplies and equipment will continue to result in a high number of casualties and infectious diseases and physical and psychological trauma will continue to negatively impact the population.

Immediate action is vital to mitigate potential disruptions and minimise the impact when the ceasefire ends, while simultaneously negotiating a permanent ceasefire remains imperative. Short‐term planning should prioritise stabilising healthcare, securing sustained safe aid routes, and stockpiling supplies for post‐ceasefire challenges. Meanwhile, long‐term planning should concentrate on infrastructure rebuilding and fostering resilience, with a focus on collaboration and community engagement.

#### 1.3.3 Scenario 3: The implementation of a permanent ceasefire

In this scenario, implementing a permanent ceasefire would be the most beneficial to the population and public health's future. With the opportunity for long‐term recovery and rebuilding as well as the opening of borders for the free flow of goods and people, sustainability and resilience can be achieved, provided that all parties involved demonstrate political will and commitment, as well as external assistance from global communities. Significant improvements will be made in terms of displacement and overcrowding will significantly improve, allowing people to be able to their place of origin and regain access to shelter and basic services, such as inadequate shelters and WASH facilities, will be restored. Malnutrition and associated health problems will be addressed by restoring food security and nutritional status. Medical facilities will have access to essential medical supplies to equipment, restoring healthcare services and trauma and injuries will cease. There will be a marked decrease in the number of casualties, control of infectious diseases, and the onset of recovery for psychological trauma and mental health issues. Implementing a permanent ceasefire would be the most beneficial to the population and public health's future. With the opportunity for long‐term recovery and rebuilding, sustainability and resilience can be achieved, provided that all parties involved demonstrate political will and commitment, as well as external assistance from global communities.

Given the dire and complex context in Gaza, a multifaceted and comprehensive approach is imperative. Immediate actions include conducting a comprehensive needs assessment and technical analysis of the main priorities in all sectors. It is crucial to acknowledge that addressing the health system cannot be done in isolation; all infrastructure and basic services must be provided. As such, swift provision of essential services including primary healthcare and field hospitals, supported by international aid for basic supplies and workforce recruitment, is crucial. All available resources must be utilised, and international intervention, especially in the initial stage, is essential. It is vital to recognise that this scenario represents the only opportunity to effectively work towards rebuilding the healthcare system in Gaza, highlighting the urgency and importance of the proposed actions within the context of a lasting ceasefire, community involvement, and the proposed roadmap for recovery that can be set into motion.

## 2 CONCLUSION

In light of this unprecedented dire crisis in Gaza, an urgent recovery strategy that entails three immediate, medium, and long‐term stages is key to addressing the profound humanitarian challenges and essential life‐saving needs. Even in the best‐case scenario of a permanent ceasefire, significant innovative and consolidated local and international efforts represented in a national Marshall recovery plan will be highly needed to rebuild the health system.

The Palestinian MOH has undertaken several health‐focused strategies to respond to the current crises, including the prioritising of emergency medical services, with over 13,000 emergency procedures performed since October 2023, amidst significant shortages in vital supplies.^21^ This urgent focus highlights the government's commitment to meeting the immediate health needs of the Palestinian population. Additionally, the Child and Adolescent Mental Health National Health Strategy (2023–2028) is another critical project launched to tackle the long‐term mental health needs of youth affected by the continuing conflict. This strategy focuses on early intervention, fostering emotional well‐being, and improving mental health care for children and adolescents who have experienced trauma.^22^ By addressing both immediate healthcare demands and long‐term challenges, it is evident that the Palestinian government is actively working to navigate pathways for recovery amidst the conflict. Moreover, one of the best strategies adopted by the MOH is operationalising the Health Cluster which is a unified collaborative platform consisting of all local and international health stakeholders in Palestine to coordinate all strategies and efforts of pathways of crisis and recovery plan. However, there is a lot of uncertainty and constant changes in the current scenes, and the MOH is advised to integrate tactical and strategic, immediate and long‐term, humanitarian and development, individual and whole society approaches in the current and post‐crisis strategies and interventions.

The proposed roadmap for recovery offers a comprehensive approach, but its success relies heavily on achieving a ceasefire and long‐lasting stability. However, regardless of the scenario, continuous efforts are imperative. Immediate humanitarian relief, stabilisation of healthcare services, and long‐term recovery efforts must proceed without delay. As we navigate these challenges, steadfast political commitment from all stakeholders is crucial. By working together with determination, collaboration, transparency, and accountability using new recovery approaches, we can strive towards restoring the health system in Gaza. Most importantly, restoring lasting health and dignity to the people of Gaza by realising the rights‐based and health‐peace approaches and ending the lengthy injustice.

## AUTHOR CONTRIBUTIONS

All authors made a significant contribution (draughting, revising, and critically reviewing the article) to the work reported. Both authors have equally contributed to the conception analysis design and article synthesis too. All authors gave final approval of the version to be published; have agreed on the journal to which the article has been submitted; and agree to be accountable for all aspects of the work.

## ETHICS STATEMENT

Not applicable.

## FINANCIAL AND NON‐FINANCIAL INTEREST

The authors have no relevant financial or non‐financial interests to disclose.

## Data Availability

Data sharing is not applicable to this article as no new data were created or analysed in this study.
